# Prof. Hufeland on the Erysipelas of New-Born Children

**Published:** 1801-06

**Authors:** 


### *Prof. Hufeland on the Erysipelas of new-born Children.*

THE Eryfipelas of new-born children is a difeafe which, on account of its rarity, is not fufficiently known to practitioners. The danger, however, with which it is attended, renders this difeafe worthy of the attention of phyficians, and its nature as well as mode of treatment, deferve to be more fully afcertained by farther refearches. We find very little mentioned on this fubject in medical books. The firft who records feveral cafes of it is Bromfield, (Med. Com.); it is likewife mentioned by Girtanner; but the beft account is given by Prof. Ofiander, of Gottingen, in his Memoirs for Phyfic and Midwifery.

The difeafe appears in the firft days of life to the fixth week, and there are only a few cafes recorded where children were born with it. Sometimes it is preceded by locked jaw and jaundice. At a fingle place, or at feveral at once, particularly in the lower extremities, regio inguinis, the neck, &c. red fpots appear; which being at firft but little raifed on the fkin, extend themfelves afterwards, whereby the parts begin to fwell, and become hard and painful. On preffing the fwelling with the finger, the fpot where the finger is applied becomes white, but without leaving a dimple. The colour of the tumour is changed, and becomes dark red and blue; gangrenous blifters and petechiæ appear; the joints affected by the difeafe become ftiff, the belly tenfe, and locked jaw and jaundice fometimes fupervene a little before death comes on. The difeafe feems in fome cafes to originate from the epidemical conftitution, at leaft Prof. Ofiander obferved it at the time when many lying-in women were affected by bilious and rheumatic fevers. The remote caufes of the difeafe are faid to be, bad diet of the mother, a corrupted atmofphere in the nurfery, colds and paffions of the mother or nurfe, as anger, fright, &c. obftructed excretion of the meconium; all which caufes feem to act upon the nerves of the belly, particularly on the organs for the fecretion of the gall, and on the lymphatic fyftem, and vafa lactea, by which the gall rendered acrimonious from the morbid irritation, is abforbed, brought to the blood, and depofited in the external parts. The prognofis is doubtful, and the ftrong or weak conftitution of the child feems to make no great difference. A fuccefsful event may be hoped according to Girtanner, ner, when the eryſipelas is confined to ſingle places, and when it begins to ſuppurate, but when it extends itſelf to a greater ſurface, and firſt appears on the belly and genitals, or when gangrene ſupervenes, the prognoſis is very bad; though Bromfield ſucceeded in ſaving a child under theſe circumſtances. It proves certainly mortal, when the inteſtines are affected, or triſmus comes on. The diſeaſe laſts from 24 hours to a fortnight, and longer.

The method of cure which has been employed here, though on the whole with little avail, conſiſts in evacuating the inteſtinal canal by vomits, and purgatives of rhubarb, manna, magneſia, and clyſters, in removing the ſpaſm by flores zinci, opium, frictions with oil, and opium applied on the belly and the liver region, in promoting diaphoreſis by antimonials, ſpiritus Mindereri, warm baths, and at the tranſition of the inflammation to gangrene, in the external and internal uſe of the Peruvian bark, camphor, &c. The application of any preparation of lead is very noxious, as by repelling the eruption, it is likely to occaſion dangerous metaſtaſes towards the inteſtines. It is likewiſe not adviſable to make uſe of the bark and camphor, when the diſeaſe is ſtill in the inflammatory ſtage; though, according to Bromfield, it may be of great ſervice, when the eryſipelas threatens to become gangrenous. It ſeems, according to what Hufeland has obſerved, that on the whole, baths of warm milk, in combination with gentle diaphoretics and antiſpaſmodics, as valerian, flores zinci, and in ſome caſes the muſk, are the beſt remedies for curing this dangerous infantile diſeaſe. On opening the body of children, who died of it, Prof. Oſiander remarked the following: The ſcrotum was ſwollen to the ſize of an egg, and at making an inciſion, a yellowiſh gelatinous matter iſſued, the veſſels were much extended by blood, the teſticles and penis not ſwollen, though their blood-veſſels were very red. The tela celluloſa between the integuments of the belly and the peritonæum contained alſo a great deal of that gelatinous matter, the veſſels being at the ſame time full of blood. The abdomen was much inflated, and filled with an orange coloured ſerum. The ſtomach was inflamed, and full of gangrenous ſpots; in one inſtance the inteſtines were much inflamed, and grown together by pſeudo membranes. Between the inteſtines a yellow puriform matter was here and there diſcovered, ſimilar to that which is found in perſons who die of hepatitis and puerperal fevers. Liver and ſpleen were likewiſe inflamed, blackiſh, and full of ſtagnant blood. The veſicula fellea contained half a drachm of black and thick gall; and, in another caſe, it was filled with a yellowiſh jelly, which was alſo contained in the vena umbilicalis. The pancreas was obdurated, and the lungs ſomewhat inflamed.

